# Iatrogenic Common Femoral Artery Occlusion Caused by a Suture-Mediated Closure System: A Case Report

**DOI:** 10.3400/avd.cr.21-00050

**Published:** 2021-12-25

**Authors:** Nobuo Kondo, Kensuke Oue, Kohei Miyashita, Satofumi Tanaka, Yoichiro Miyake

**Affiliations:** 1 Department of Cardiovascular Surgery, Kochi Health Science Center, Kochi, Kochi, Japan

**Keywords:** CFA occlusion, suture-mediated closure system, Perclose ProGlide

## Abstract

Vascular closure devices (VCDs) are useful for reducing bed rest time after percutaneous catheterization procedure without manual compression at the femoral puncture site. Occlusion of the common femoral artery (CFA) related to VCDs has rarely been reported. Although catheter treatment for CFA occlusion may be the first choice, it may be insufficient. Surgical treatment should be performed immediately when catheter treatment for artery occlusion is deemed difficult. We report a case of surgical angioplasty performed for femoral artery occlusion by using a suture-mediated device.

## Introduction

Vascular closure devices (VCDs) are useful for reducing bed rest time after percutaneous catheterization procedure without manual compression at the femoral puncture site. One of the VCDs is a suture-mediated closure device (Perclose ProGlide; Abbott Vascular, Inc., Redwood City, CA, USA). In Japan, ProGlide has been applicable to insurance since 2020. Nikolsky et al. reported that the vascular closure system for the femoral artery has been widely used for achieving better hemostasis and reducing complications compared with manual compression.^1,2)^

The rates of VCD complications, such as hematoma, bleeding, pseudoaneurysm, thrombosis, and infection, are reported to be 0% to 6%.^1)^ Common femoral artery (CFA) stenosis or occlusion is rarely reported, especially in Japan.^3–6)^

Herein, we report a case of femoral artery occlusion with claudication caused by ProGlide and treated with surgical angioplasty.

## Case Report

**Figure 1:**
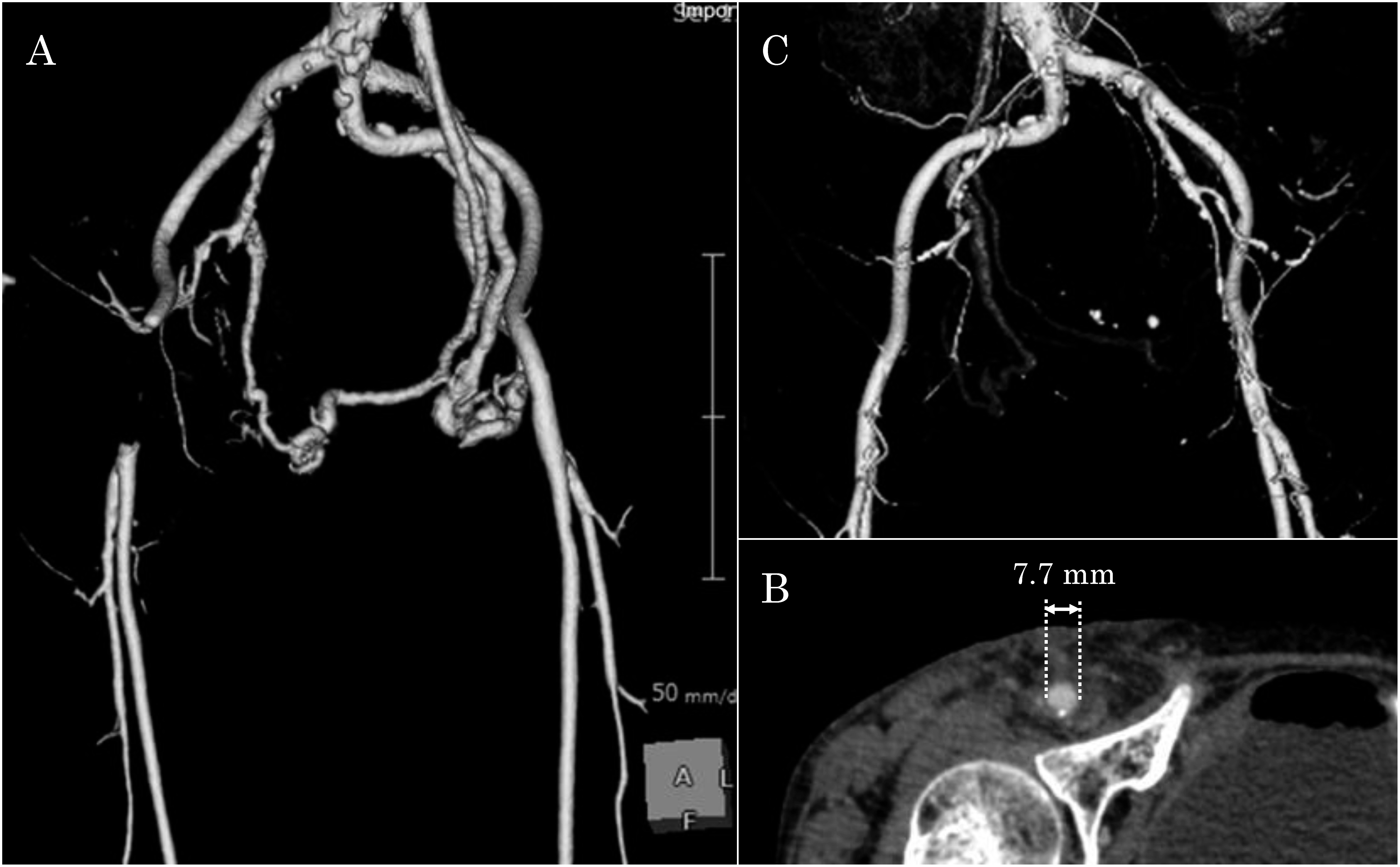
Fig. 1 Contrast-enhanced computed tomography (CT) images. (**A**) and (**B**) are the preoperative 3-dimensional (3D)CT and axial CT images. (**A**) shows the occlusion of the right common femoral artery (CFA) and thrombus from the right external iliac artery to the CFA. (**B**) demonstrates that the diameter of the CFA is 7.7 mm. (**C**) is the postoperative 3DCT and shows that the anastomosis has no stenosis.

A 68-year-old woman underwent coil embolization for an unruptured cerebellar aneurysm using a retrograde approach via the right CFA with a 7 Fr system at another hospital. The coil embolization was successful. A suture-mediated closure device (Perclose ProGlide) was used for hemostasis of the right CFA. Hemostasis was longer than expected. The following day, the patient complained of right-leg pain and a cold feeling at rest. Her height and body weight were 152 cm and 33.7 kg, respectively. Physical examination revealed a blood pressure of 153/91 mmHg, regular pulse rate of 89 beats/min, body temperature of 37.9°C, and oxygen saturation of 97% in room air. The right dorsalis pedis artery pulse was not detected at this time. Blood examination revealed an inflammatory response (white blood cell 11650/µL, C-reactive protein 0.99 mg/dL), elevated levels of creatine kinase (589 U/L), and fibrinolysis (D-dimer 3.0 µg/mL). Contrast-enhanced computed tomography (CT) revealed total occlusion of the right CFA and thrombus from the right external iliac artery to the CFA (**Fig. 1A**). This indicated that the diameter of her right CFA was 7.7 mm (**Fig. 1B**). Because the CFA is the so-called non-stenting zone, we performed surgical angioplasty.

**Figure 2:**
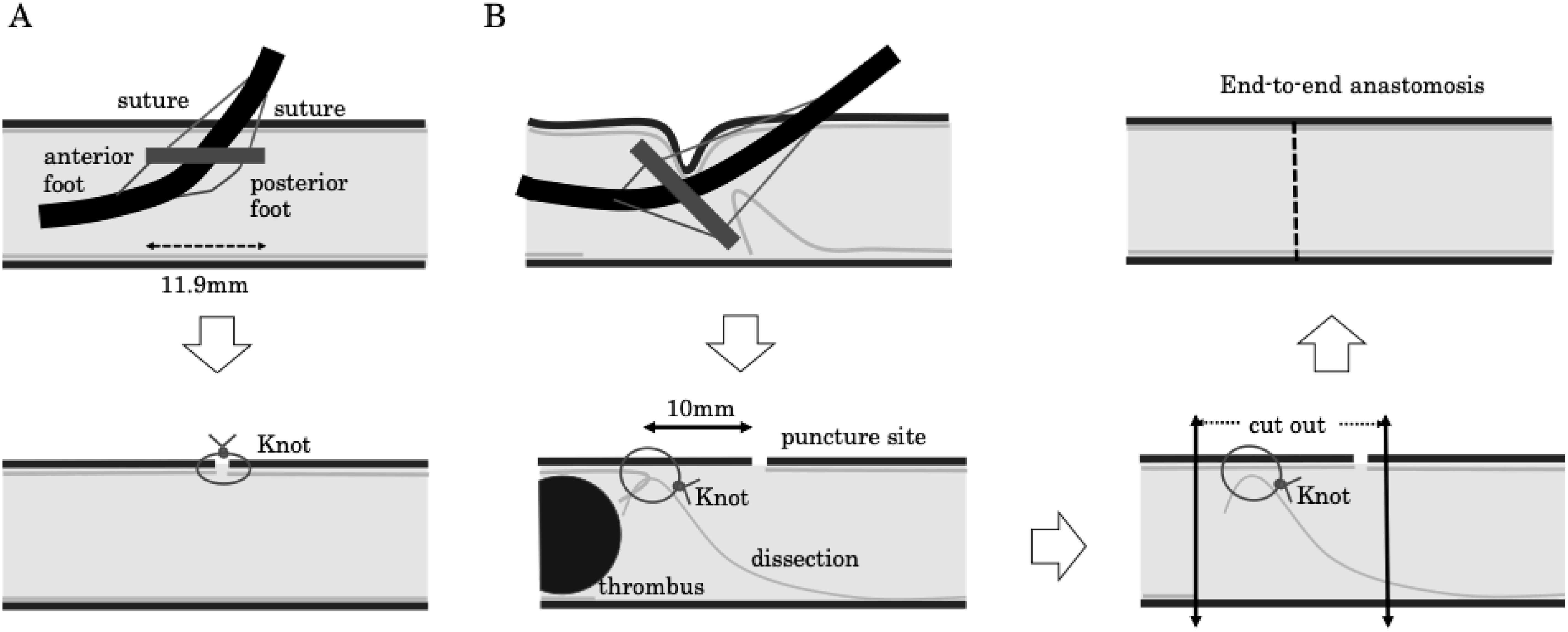
Fig. 2 Illustration of the common femoral artery (CFA) occlusion. (**A**) shows the feet length and the proper deployment of ProGlide, which is positioned with both the anterior and posterior feet against the anterior wall of the CFA. (**B**) shows an illustration of the occlusion mechanism and the operation. Because the puncture angle is less than 45°, the anterior foot stitched at the anterior arterial wall twice, and the posterior foot dissected the posterior arterial wall. The torn artery was cut out and underwent end-to-end anastomosis.

Under local anesthesia, the right CFA was approached via a surgical cut-down. Although the puncture site was detected on the CFA with hematoma, the knot of ProGlide was not detected at the same site. After the CFA clamp and incision of the same site, the knot of ProGlide was detected inside the CFA on 1 cm head from the puncture site. The entire layer of the anterior wall and the intima of the posterior wall were stitched. In addition, the arterial intima was torn, and a large amount of thrombus was detected on the central side (**Fig. 2B** lower left). Subsequently, the native vessel underwent end-to-end anastomosis after thrombectomy was performed. A large amount of red blood clot was removed (**Fig. 2B** right).

After surgery, both the limb symptoms and blood examination improved simultaneously. The postoperative CT assessment on the 5th day demonstrated no problem with the anastomosis (**Fig. 1C**). The postoperative course was uneventful, and the patient was discharged at 6 days following surgery. Informed consent was obtained from the patient for the publication of this case report and the accompanying images (Clinical trial registration number: 21005).

## Discussion

The Perclose ProGlide suture-mediated closure device system is indicated for percutaneous access close to the CFA and vein in patients who undergo catheterization. In Japan, ProGlide is applicable to insurance by 2020. ProGlide has two pairs of non-absorbable polyester sutures and is indicated for 5–21 Fr femoral arteriotomy sites. ProGlide is inserted over a wire and advanced until blood flow is observed to identify positioning within the vascular lumen. The foot is deployed within the artery and delivers the needles to the puncture site (**Fig. 2A**).^1,7,8)^ In addition, it is important to place the foot in close contact with the anterior arterial wall by pulling up the device.^7,8)^ In this case, the anterior and posterior feet of ProGlide were deployed each against the anterior and posterior walls of the CFA, respectively. Because the device was pulled at a shallow angle, the feet of the device deformed the CFA and blocked the blood flow. This was considered to be the cause of the misunderstanding that the feet were in close contact with the anterior vessel wall. As a result, the entire layer of the anterior wall and the intima of the posterior wall were stitched (**Fig. 2B** upper left). It was considered to be caused by the pulling of the device at an angle less than 45°. The foot length and angle of the device were 11.9 mm and 45°, respectively. The diameter of the patient’s CFA was 7.7 mm. Therefore, the puncture angle should be carefully considered when the arterial diameter is small. When the puncture angle is less than 45°, the foot may block the vessels and blood flow. In many cases with complications, the operator felt a normal tactile feedback and observed disappearing pulsatile blood from the marker lumen to ensure that the foot is in the proper position. Therefore, it is important to check the presence of claudication and the dorsalis pedis artery pulse after using ProGlide.^2,7,8)^

The treatments of stenosis or occlusion with the use of ProGlide is reported to be surgical angioplasty and catheter treatment with balloon angioplasty or stent implantation.^3–6,8)^ Although there is no consensus on the treatment of occlusion using VCDs, there are many reports about successful catheter treatment.^3,4,6,8)^ Catheter treatment may be the first choice. However, balloon angioplasty is associated with a risk of residual stenosis and dissection. Moreover, stent implantation at CFA, which is the so-called non-stenting zone, carries a high risk of stent fracture because of the constant flection at the inguinal ligament.^2,8)^ When catheter treatment is uncertain, surgical repair should be performed. In our case, the CFA was completely occluded by thrombus. Because catheter treatment was associated with a high risk of residual stenosis, embolism, and vessel tear, we performed surgical repair under local anesthesia without complications. Since occlusion by the ProGlide occurs through various mechanisms, it is important to perform surgical treatment even after catheter treatment. Hereafter, although the rate of complications with ProGlide does not change, the number of them will increase with the spread of using VCDs.

Treatment of CFA occlusion using VCDs such as ProGlide may be better with surgical repair than with catheter treatment.

## Conclusion

Herein, we report a case of CFA occlusion related to ProGlide. ProGlide is useful for hemostasis after catheter treatment. Although CFA occlusion is rare, it may occur. Therefore, we should consider the use of ProGlide; it should also be used carefully. In addition, the pulsation of the peripheral artery should be checked after treatment. When catheter treatment for CFA occlusion related to ProGlide is deemed difficult, surgical angioplasty should be performed at the earliest.
